# Clinical Investigations of CAR-T Cell Therapy for Solid Tumors

**DOI:** 10.3389/fimmu.2022.896685

**Published:** 2022-07-18

**Authors:** Kun Chen, Shuhang Wang, Dan Qi, Peiwen Ma, Yuan Fang, Ning Jiang, Erxi Wu, Ning Li

**Affiliations:** ^1^ National Health Commission (NHC) Key Laboratory of Pulmonary Immune-Related Diseases, Guizhou Provincial People’s Hospital, Guiyang, China; ^2^ Clinical Cancer Center, National Cancer Center/National Clinical Research Center for Cancer/Cancer Hospital, Chinese Academy of Medical Sciences and Peking Union Medical College, Beijing, China; ^3^ Department of Neurosurgery and Neuroscience Institute, Baylor Scott & White Health, Temple, TX, United States; ^4^ Texas A&M University Colleges of Medicine and Pharmacy, College Station, TX, United States; ^5^ LIVESTRONG Cancer Institutes and Department of Oncology, Dell Medical School, The University of Texas at Austin, Austin, TX, United States

**Keywords:** cellular immunotherapy, chimeric antigen receptors, genetically engineered, tumor microenvironment, solid tumors

## Abstract

Cell therapy is a distinguished targeted immunotherapy with great potential to treat solid tumors in the new era of cancer treatment. Cell therapy products include genetically engineered cell products and non-genetically engineered cell products. Several recent cell therapies, especially chimeric antigen receptor (CAR)-T cell therapies, have been approved as novel treatment strategies for cancer. Many clinical trials on cell therapies, in the form of cell therapy alone or in combination with other treatments, in solid tumors, have been conducted or ongoing. However, there are still challenges since adverse events and the limited efficacy of cell therapies have also been observed. Here, we concisely summarize the clinical milestones of the conducted and ongoing clinical trials of cell therapy, introduce the evolution of CARs, discuss the challenges and limitations of these therapeutic modalities taking CAR-T as the main focus, and analyze the disparities in the regulatory policies in different countries.

## Introduction to Cell Therapy

While traditional cytotoxic agents still play important roles as one of the critical mainstays in therapeutics for malignant solid tumors, targeted small-molecule drugs, antibodies, and cell therapies are emerging as new directions. Indeed, cell therapies have recently helped transform the treatment blueprint for cancer patients. Cell therapy is generally considered as the transplantation of autologous or allogeneic cellular material into a patient for therapeutic purposes (1). Cell therapy products include genetically engineered cell products and non-genetically engineered cell products. Cell therapy has been regarded as “the third pillar of future medicine” with great potential to treat solid tumors in the new era of cancer treatment (2).

At present, the subcategories of cell therapy products used to treat tumors are as follows: engineered chimeric antigen receptors (CARs), T-cell receptor-engineered T-cell therapy (TCR-T), tumor-infiltrating lymphocytes (TILs), tumor vaccines, stem cells, dendritic cells mixed with cytokine-induced killer (DC-CIK) cells, CIK cells, and natural killer (NK) cells. CARs and TCR-T are parts of specific immune therapy modified by genetic engineering, which have attracted much attention because of their excellent therapeutic effects. Two CAR-T therapies—Kymriah (3) and Yescarta (4)—were approved by the United States (US) Food and Drug Administration (FDA) in 2017, with objective remission rates of 83% (52/63) and 72% (73/101), respectively, for recurrent or refractory B-cell acute leukemia. In 2020, the FDA approved Tecartus (5), the first cell-based gene therapy for relapsed or refractory mantle cell lymphoma, with an objective remission rate of 87% (52/60) and a complete response of 62% (37/60). Most recently, ABECMA (idecabtagene vicleucel) (6) was approved by the FDA for adult patients with relapsed or refractory multiple myeloma after four or more prior lines of therapy in 2021. The National Medical Products Administration (NMPA) approved FKC876, the first cell-based gene therapy for relapsed or refractory indolent non-Hodgkin’s lymphoma in China. These demonstrate the current success of cell therapy, including CAR-T therapy, for blood cancers. However, cell-based therapies are not yet quite effective in treating solid tumors due to heterogeneous antigens in solid tumors, complex microenvironment (7–9), limited targetable antigens (10), difficulties in immune cell migration, and tumor infiltration (11, 12). Despite these difficulties, some cell therapies have already been approved by the FDA for clinical practice in solid tumors. For example, the use of sipuleucel-T, a dendritic cell (DC) vaccine (13), and its related clinical trials have been reported in recent years. Furthermore, the global market value of cell therapy technology has rapidly grown and is expected to exceed $34 billion by 2025 (14). Thus, cell therapies would be a huge leap from traditional therapies.

Cell therapy is closely related to the fields of targeted therapy, gene therapy, and regenerative medicine, making it even more complicated in clinical practice. Meanwhile, cell therapy has become an independent evaluation system different from traditional drugs, especially with regard to the assessment of safety and efficacy. Therefore, the management system of cell therapy varies worldwide (**Table 1**). Here, we provide a concise overview of cell therapy, especially CAR-T therapy; enumerate the relevant clinical trials initiated in the last decade; and summarize the disparities in regulatory policies in different countries. In addition, we assess the conceptual framework and specific therapeutic strategies as well as the challenges and limitations of CAR-T therapies.

**Table 1:** Government policies regarding cell therapies worldwide.

Region	Agency in charge	Government policy	Year of promulgation	Definition	Classification	Reference
European Union	EMA (European Medicines Agency)	Guideline on Human Cell-Based Medicinal Products (Doc. Ref. EMEA/CHMP/410869/2006)	2008	The European Medicines Agency’s scientific guidelines on cell therapy and tissue engineering help medicine developers prepare marketing authorization applications for human medicines. Cellular therapy products fall into the category of advanced therapy medicinal products.	Human cell-based medicinal products are heterogeneous with regard to the origin and type of the cells and to the complexity of the product. Cells may be self-renewing stem cells, more committed progenitor cells, or terminally differentiated cells exerting a specific defined physiological function. Cells may be of autologous or allogeneic origin. Cells may also be genetically modified. Cells may be used alone, associated with biomolecules or other chemical substances, or combined with structural materials that alone may be classified as medical devices.	https://www.ema.europa.eu/en/human-cell-based-medicinal-products
Japan	PMDA (Pharmaceuticals and Medical Devices Agency)	Act on Securing Quality, Efficacy and Safety of Pharmaceuticals, Medical Devices, Regenerative and Cellular Therapy Products, Gene Therapy Products, and Cosmetics (abbreviated as the PMD Act)	2014	PMDA offers consultations to give guidance and advice on clinical trials of drugs, medical devices, and cellular and tissue-based products as well as on data for regulatory submissions. Cellular therapy products fall into the category of regenerative medical products.	Products intended for use in human or animal healthcare, which are obtained after culturing or other processes using human or animal cells. Products intended for use in the treatment of disease in humans or animals, which are introduced into the cells of humans or animals and contain genes to be expressed in their bodies.	http://www.japaneselawtranslation.go.jp/law/detail_main?re=&vm=2&id=3213
United States	FDA-CBER (The Center for Biologics Evaluation Research)	Considerations for the Design of Early-Phase Clinical Trials of Cellular and Gene Therapy Products Guidance for Industry (FDA-2013-D-0576-0019)	2015	CBER regulates cellular therapy products, human gene therapy products, and certain devices related to cell and gene therapy. CBER uses both the Public Health Service Act and the Federal Food Drug and Cosmetic Act as enabling statutes for oversight.	Cellular therapy products include: Cellular immunotherapies Cancer vaccines Other types of both autologous and allogeneic cells for certain therapeutic indications Hematopoietic stem cells and adult and embryonic stem cells	https://www.fda.gov/vaccines-blood-biologics/cellular-gene-therapy-products
China	CFDA (China Food and Drug Administration)	Technical Guidelines for the Research and Evaluation of Cell Therapy Products (Trial version, 2017-NO216)	2017	Human-derived alive cell products to treat human diseases are investigated, developed, and registered in accordance with the policy of drug administration. The source, processing, and investigating clinical trials of these products meet the ethics.	Cell therapy products derived from human cells are used to treat human diseases. Excluding: Blood components used for blood transfusions Hematopoietic stem cell transplantation without *in-vitro* treatment Reproductive cells Tissues or organs	http://www.nmpa.gov.cn/WS04/CL2138/300457.html

## Milestones of Cell Therapy for Solid Tumors

**Figure 1:**
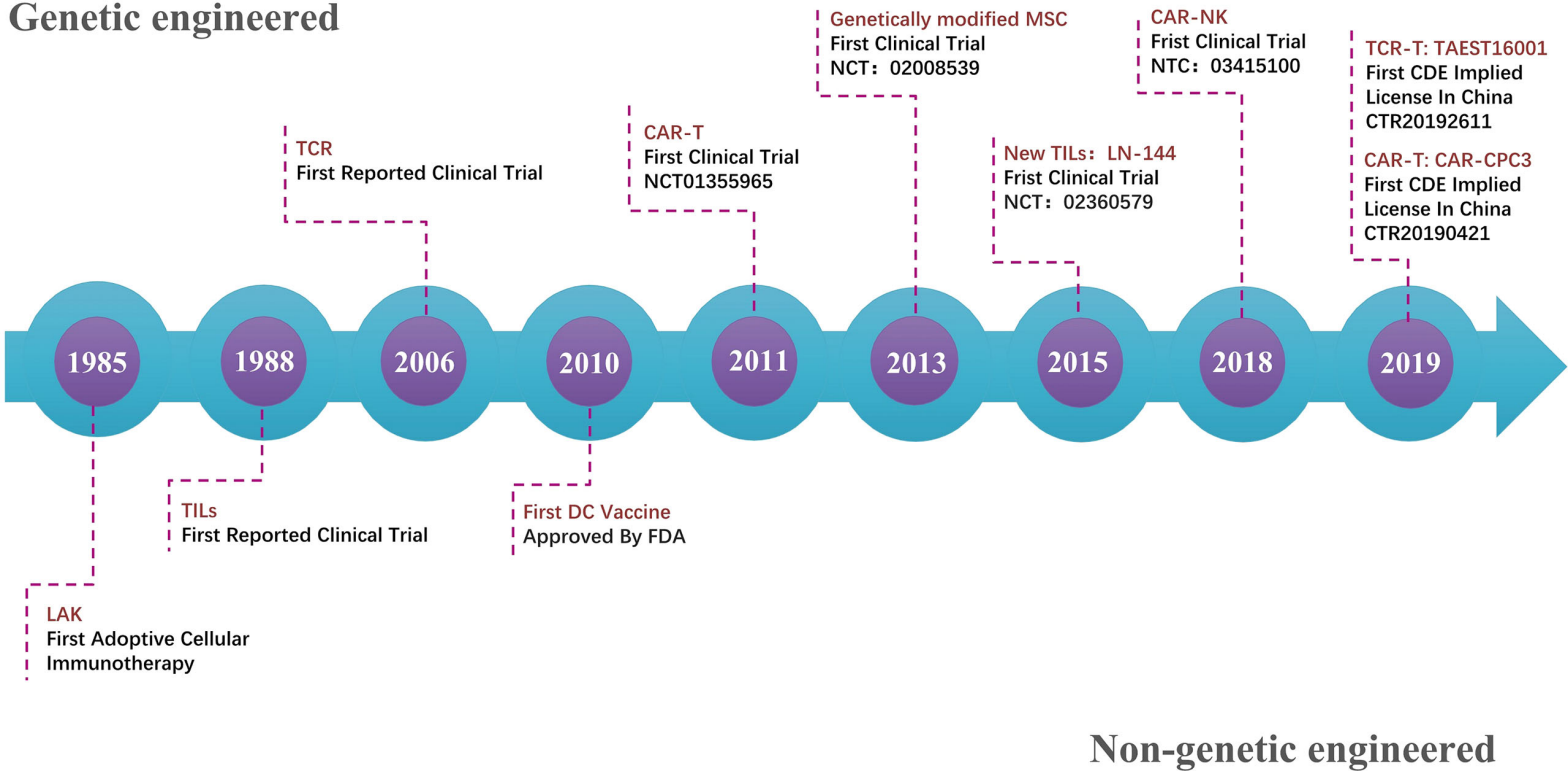
Timeline of important events for cell therapy in solid tumors. After the first adoptive cellular immunotherapy, LAK opened the gate of cell therapy in 1985. Cell therapy has rapidly evolved in the last 10 years with a consistent appearance of novel types. The transformation from the non-genetic engineering model to genetic engineering-based products is the most important direction.

Important events of cell therapy in solid tumor treatment are summarized in **Figure 1**. The first adoptive cellular immunotherapy, lymphokine-activated killer (LAK) cells (15), opened the door for cell therapy. The safety of using high-dose interleukin (IL)-2 is still an issue, although it has a very broad spectrum antitumor effect. In 1988, the first reported clinical trial of TILs showed a 60% objective regression rate (16). In 2006, the first study using TCR reported a substantial sustainable regression (17). In 2010, the first DC vaccine was approved by the FDA for metastatic, asymptomatic hormone-refractory prostate cancer (13) with a 3-year survival rate of 34%. In 2013, the first trial of genetically modified MSCs (MSC_apceth_101) was initiated (NCT02008539) (18). Five out of 10 patients treated with MSC_apceth_101 achieved stable disease. The combination of MSC_apceth_101 with ganciclovir was reported to be safe and tolerable in patients with advanced gastrointestinal adenocarcinoma (19, 20). In 2015, the first trial of genetically modified TILs (LN-144) was initiated (NCT02360579). From the current result, LN-144 treatment results in an overall response rate of 36.4% and a median duration of response not reaching 17 months of median follow-up time in metastatic melanoma patients who progressed on multiple prior therapies (21). In 2018, the first clinical trial using CAR-NK was initiated (NCT03415100), which is still ongoing. In 2019, the first TCR-T and CAR-T (CAR-glypican-3) trial in China obtained the CDE license.

## Official Government Policies for Cell Therapy Worldwide

The available official regulatory policies around the world regarding cell therapies, including those in the USA, Europe, Japan, and China, were collected, reviewed, and summarized (**Table 1**).

In September 2008, the guideline on Human Cell-Based Medicinal Products from the Committee for Medicinal Product for Human Use of the Europe Medicines Agency came into effect. It defined cell therapy as cell-based medical products, including self-renewing stem cells, autologous or allogeneic origin cells, and genetically modified cells. The present document applies only to the cellular component of the cell-based medicinal products containing genetically modified cells.

In November 2014, the Pharmaceuticals and Medical Devices Agency (PMDA) of Japan published “Act on Securing Quality, Efficacy and Safety of Pharmaceuticals, Medical Devices, Regenerative and Cellular Therapy Products, Gene Therapy Products, and Cosmetics.” The Act defined cell therapy products as those intended for use in human or animal healthcare, which are obtained after culturing or other processes using human or animal cells, as well as those intended for use in the treatment of disease in humans or animals, which are introduced into the cells of humans or animals and contain genes to be expressed in their bodies.

In June 2015, the Center for Biologics Evaluation and Research (CBER)/Office of Cellular, Tissue, and Gene Therapies of the USFDA issued “Considerations for the Design of Early-Phase Clinical Trials of Cellular and Gene Therapy Products – Guidance for Industry” (FDA-2013-D-0576). This guidance defined cellular therapy products as cellular immunotherapies, cancer vaccines, and other types of both autologous and allogeneic cells for certain therapeutic indications. It also mentioned that the risk and safety problems associated with specific types of cell therapy products should be carefully considered based on limited experiences. Furthermore, it gives specific recommendations and evaluation criteria for a wide range of situations/settings, including manufacturing, preclinical use, clinical trial design, dose determination, and assessment of feasibility and activity of therapy, among others. In 2016, CBER launched a new policy aimed at accelerating the review of cell therapy products for serious or life-threatening diseases, called the “Regenerative Medicine Advanced Therapy Designation,” to guarantee the availability of novel products for patients who most urgently need them. Afterward, the USFDA released “Expedited Programs for Regenerative Medicine Therapies for Serious Conditions” (FDA-2017-D-6159), which further clarifies that products defined as fast track, breakthrough therapy, or regenerative medicine advanced therapy (RMAT) designation are able to go through priority review and accelerated approval. Due to the current coronavirus pandemic, the USFDA (FDA-2020-D-1137) has recently released “Manufacturing Considerations for Licensed and Investigational Cellular and Gene Therapy Products During COVID-19 Public Health Emergency,” highlighting the importance to avoid virus contamination from various sources such as cellular and tissue samples from donors.

In December 2017, China’s FDA [CFDA, now known as the National Medical Product Administration (NMPA)] published the “Technical Guidelines for Research and Evaluation of Cell Therapy Products (Trial version, 2017-NO216).” It describes cell therapy as “products derived from human cells that are used to treat human diseases.” This is a trial version asking for professional inputs, as formal guidance is in urgent need. A “double-track system” is currently used by the Chinese authorities for the oversight of cell therapy products, comprising the drug “registration system” of The Center for Drug Evaluation (CDE) under NMPA and the medical technology “filing system” of the National Health Commission. Many clinical trial studies are carried out through the “filing system,” which has relatively loose requirements, rather than through the “registration system” with relatively strict requirements for investigational new drug (IND) research. This practice has promoted the development of cell therapy products and industry in clinical application. However, more clinical trials of cell therapy were registered on www.clinicaltrials.gov than those registered as IND in CDE. Now, updated guidelines are called, considering the rapid development of innovative pharmaceutical enterprises in China.

To sum up, cell therapy is an interdisciplinary field with complex safety concerns and efficacy standards, which greatly differ from those of traditional drugs, leading to the divergence in definition and administration patterns as summarized in **Table 1**. It is believed that a refinement of the documents is a pressing need in order to efficiently share, manage, and oversee cell-based products in clinical trials and medical practice. Meanwhile, the rapid research advancement requires a continuously updated management of cell therapy products to ensure both innovation and safety.

## Trends of Investigational Products on Cell Therapy

### Conducted and Ongoing Clinical Trials During the Past Decade

The clinical trials of cell therapies carried out from January 1, 2011, to January 1, 2021, worldwide were derived and analyzed from the Pharmaprojects database (https://citeline.informa.com/trials/) developed by a leading international research group known as INFORMA (**Supplementary Information**) to illustrate the status of clinical investigations of cell therapy in solid tumors both worldwide and specifically in mainland China.

A total of 572 clinical trials were recorded, among which 112 trials (19.6%) were CAR-related, 87 (15.2%) were on TCR-T, 17 (3.0%) on stem cells, 188 (32.8%) on cell-based vaccines, and 168 (29.4%) on others (on TILs, DC-CIK, CIK, and NK cells) (**Table 2**). Most of the trials are phase 1 or phase 1/2 (370/572, 64.7%), and only 24 (4.2%) trials are phase 3. Among those, 198 (34.6%) have been completed; however, only three products investigated in these trials have been approved, namely, sipuleucel-T (22) by the USFDA for treating asymptomatic or minimally symptomatic metastatic castrate-resistant (hormone-refractory) prostate cancer; autologous dendritic cells by the Indian FDA (23) for prostate, ovarian, colorectal, and non-small cell lung carcinomas; and Immuncell-LC in South Korea for liver cancer (24) worldwide.

**Table 2:** Classification and characteristics of clinical trials on cell therapy worldwide.

Items	Type	CAR *n* (%)	TCR *n* (%)	Stem *n* (%)	Vaccine *n* (%)	Other *n* (%)	Total *n* (%)
Phase	I	78 (69.6)	44 (50.6)	8 (47.1)	49 (26.1)	49 (29.2)	228 (39.9)
	I/II	30 (26.8)	28 (32.2)	4 (23.5)	46 (24.5)	34 (20.2)	142 (24.8)
	II	3 (2.7)	14 (16.1)	5 (29.4)	76 (40.4)	80 (47.6)	178 (31.1)
	III/IV/other	1 (0.9)	1 (1.1)	0	17 (9.0)	5 (3)	24 (4.2)
Trial status	Open	71 (63.4)	37 (42.5)	11 (64.7)	38 (20.2)	58 (34.5)	215 (37.6)
	Closed	18 (16.1)	26 (29.9)	4 (23.5)	57 (30.3)	54 (32.1)	159 (27.8)
	Completed	23 (20.5)	24 (27.6)	2 (11.8)	93 (49.5)	56 (33.3)	198 (34.6)
	Totally (*n*/572*%)	112 (19.6)	87 (15.2)	17 (3.0)	188 (32.8)	168 (29.4)	572 (100)
Primary endpoint	Safety	96 (85.7)	66 (75.9)	13 (76.5)	98 (52.1)	87 (51.8)	360 (62.9)
	Efficacy	16 (14.3)	21 (24.1)	4 (23.5)	90 (47.9)	81 (48.2)	212 (37.1)
Patient population	Biomarker selected	30 (26.8)	36 (41.4)	0	12 (6.4)	18 (10.7)	96 (16.8)
	Unselected	82 (73.2)	51 (58.6)	17 (100)	176 (93.6)	150 (89.3)	476 (83.2)
Area	China	60 (53.6)	20 (23.0)	2 (11.8)	6 (3.2)	52 (31.0)	140 (24.5)
	USA	42 (37.5)	57 (65.5)	9 (52.9)	116 (61.7)	55 (32.7)	279 (48.8)
	Europe	6 (5.4)	2 (2.3)	3 (17.6)	39 (20.7)	21 (12.5)	71 (12.4)
	Other	4 (3.6)	8 (9.2)	3 (17.6)	27 (14.4)	40 (23.8)	82 (14.3)
Therapy method	Neoadjuvant	3 (2.7)	2 (2.3)	0	15 (8.0)	3 (1.8)	23 (4.0)
	Adjuvant	6 (5.4)	5 (5.7)	0	42 (22.3)	18 (10.7)	71 (12.4)

NA, not applicable/available.

In China, 140 trials have been initiated in the last 10 years, consisting of 60 (42.9%) trials on CAR, 20 (14.3%) on TCR-T, 6 (4.3%) on vaccine, 2 (1.4%) on stem cells, and 52 (37.1%) on others, none of which have been approved in China yet. Phase 1 and phase 1/2 trials account for 75.7% (105/140), phase 2 trials account for 20.7% (29/140), and only 5 (3.6%) trials are phase 3 (**Table 3**).

**Table 3:** Classification and characteristics of clinical trials on cell therapy in China.

Items	Classification	CAR *n* (%)	TCR *n* (%)	Vaccine *n* (%)	Stem *n* (%)	Other *n* (%)	Total *n* (%)
Phase	I	36 (60.0)	16 (80.0)	1 (16.7)	0	12 (23.1)	65 (46.4)
	I/II	22 (36.7)	3 (15.0)	1 (16.7)	1 (50.0)	14 (26.9)	41 (29.3)
	II	2 (3.3)	1 (5.0)	3 (50.0)	1 (50.0)	22 (42.3)	29 (20.7)
	III/IV/other	0	0	1 (16.7)	0	4 (7.7)	5 (3.6)
Trial status	Open	40 (66.7)	13 (65.0)	2 (33.3)	1 (50.0)	18 (34.6)	74 (52.8)
	Closed	6 (10.0)	1 (5.0)	0	0	12 (23.1)	19 (13.6)
	Completed	14 (23.3)	6 (30.0)	4 (66.7)	1 (50.0)	22 (42.3)	47 (33.6)
	Totally (*n*/140*%)	60 (42.9)	20 (14.3)	6 (4.3)	2 (1.4)	52 (37.1)	140 (100)
Tumor types top 5	Liver	12 (20.0)	9 (45.0)	4 (66.7)	0	12 (23.1)	37 (26.4)
	Pancreas	9 (15.0)	3 (15.0)	1 (16.7)	0	17 (32.7)	30 (21.4)
	Lung	15 (25.0)	0	3 (50.0)	0	9 (17.3)	27 (19.3)
	Gastric	7 (11.7)	3 (15.0)	1 (16.7)	1 (50.0)	7 (13.5)	19 (13.6)
	Esophageal	14 (23.3)	0	0	0	4 (7.7)	18 (12.9)
	Unspecified solid tumor	11 (18.3)	3 (15.0)	1 (16.7)	0	4 (7.7)	19 (13.6)
Primary endpoint	Safety	48 (80.0)	17 (85.0)	2 (33.3)	1 (50.0)	16 (30.8)	84 (60.0)
	Efficacy	12 (20.0)	3 (15.0)	4 (66.7)	1 (50.0)	36 (69.2)	56 (40.0)
Patient population	Biomarker selected	13 (21.7)	7 (35.0)	0	0	7 (13.5)	8 (19.3)
	Unselected	47 (78.3)	13 (65.0)	6 (100)	2 (100)	45 (86.5)	113 (80.7)

NA, not applicable/available.

**Figure 2:**
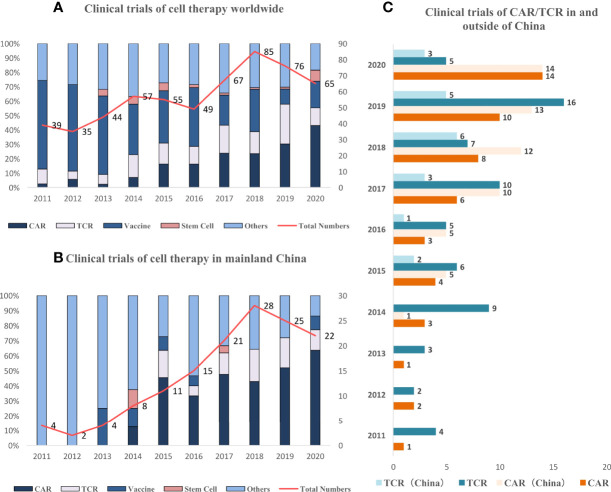
Trend of clinical trials on cell therapy worldwide and in China. **(A)** Newly initiated active clinical trials on cell therapy have increased especially after 2014, and the dominating type is converting from vaccine to CAR/TCR-T. **(B)** Newly initiated active clinical trials in China continue to increase since 2015, and the dominating type is CAR/TCR. **(C)** Newly initiated active clinical trials increase both in and outside of China (since 2015).

Most of the clinical trials on cell therapy are in the early stage of evaluating safety instead of efficacy both worldwide (360, 62.9%) and in China (84, 60.0%). The abundance rate for products with specific targets, such as TCR-T and CAR-T, was 34.8% (199/572) worldwide, which was similar to that in China (57.2%, 80/140). The recruited patient population comprised mostly those with stage III to IV solid tumors in second- or later-line treatment both worldwide and in China. The proportion of biomarker-selected trials on these patients was still low both worldwide and in China (16.8% and 19.3%). The newly initiated clinical trials have increased both in and outside of China (**Figures 2A, B**).

The landscape of targets in clinical trials was also different between China and the rest of the world. The top 3 targets of CARs were mesothelin, GPC3, and mucin-1 (MUC1) worldwide and GPC3, MUC1, and Claudin18.2 in China. The top 3 targets of TCR-T were NY-ESO-1, pHLA, and MAGE-4 worldwide. NY-ESO-1, HBV, and HPV were the top 3 targets in China, which was dominated by virus-specific targets. This abundance was much higher compared with that in other regions excluding China, indicating that the incidence of these distinctive virus-specific tumors in China was still high. The clinical trials on CAR-T and TCR-T have increased both inside and outside of China during the last 10 years, especially after 2017 (**Figure 2**).

The cell therapy of solid tumors has emerged as a novel strategy with promising efficacy data, but it is still under early-phase trial investigations worldwide, including in China.

## CAR Therapy for Solid Tumors

**Figure 3:**
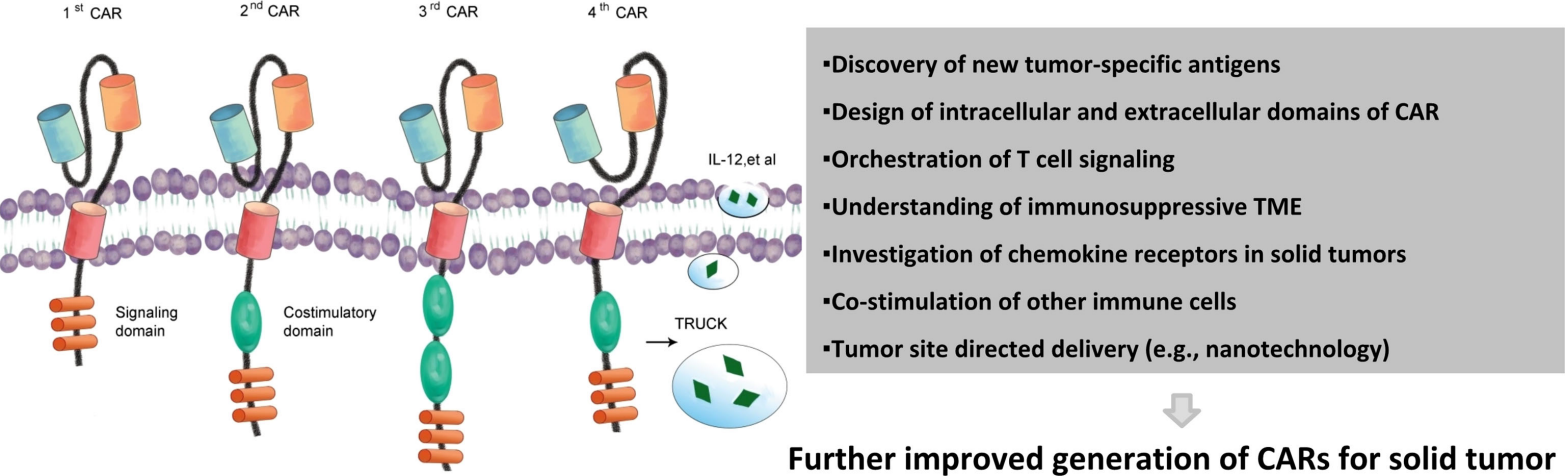
Chimeric antigen receptors and main challenges of treating solid tumors. Evolution of CAR from the first generation to the fourth generation. First-generation CARs. Second-generation CARs embody an additional intracellular signaling domain to the first-generation receptor configuration and provide a co-stimulatory signal. Third-generation receptors incorporate two co-stimulatory domains with the T-cell-activating signaling domain. Fourth-generation CARs or TRUCKS (T cells redirected for universal cytokine killing) carry vectors that encode a CAR and a CAR-responsive promoter as well as respond to the successful signaling of CAR by the transgenic production of cytokines such as IL-12. Upon accumulated knowledge of solid tumors and their surrounding environment, more effective CAR therapeutic products may be generated. CAR, chimeric antigen receptors.

More than half of all cell therapy trials in China are CAR-based; hence, the use of the most promising subtype of cell therapies, CAR, in treating solid tumors was discussed hereafter. CAR is a synthetic molecule comprising an extracellular domain, a transmembrane domain, and an intracellular signaling domain (**Figure 3**) (25). CAR-T immunotherapy has achieved remarkable success in treating hematologic cancers (26). Unfortunately, the clinical findings of CAR in treating solid tumors have been discouraging so far.

### Challenges of CARs for Solid Tumors

Identifying antigens exclusively expressed on tumor cells is the key factor for effective CAR-T exploration in solid tumors, which has been most challenging. A growing number of clinical trials now focus on solid tumors, targeting tumor-associated antigens (TAAs) including disialoganglioside GD2 (e.g., NCT03721068, NCT04637503) (27–30), human epidermal growth factor receptor 2 (HER2) (e.g., NCT04650451, NCT03740256) (31), epidermal growth factor receptor variant III (EGFRvIII) (e.g., NCT02844062, NCT03423992) (31–33), carcinoembryonic antigen (CEA) (e.g., NCT04348643, NCT04513431) (28), interleukin (IL) 13Rα2 (e.g., NCT04003649), prostate-specific membrane antigen (PSMA) (e.g., NCT04227275, NCT04249947) (34, 35), neural cell adhesion molecule L1 (NCAM-L1, CD171) (e.g., NCT02311621) (36), receptor tyrosine kinase-like orphan receptor 1 (ROR1) (e.g., NCT02706392) (37), mesothelin (e.g., NCT03545815, NCT03054298) (38, 39), and B7-H3 (e.g., NCT04185038, NCT04483778, NCT04077866) (40). Another potential immunotherapy target for solid tumors is tumor necrosis factor (TNF)-related apoptosis-inducing ligand (TRAIL) death receptors. The TRAIL ligand receptors, death receptors 4 (DR4)/DR5, are widely expressed in many cancer cells, but not expressed in normal tissues (41, 42). Importantly, on-target/off-tumor toxicities to normal cells attributable to cross-reactivities have not been observed for the aforementioned antigens.

Inadequate antigen expression to trigger optimal CAR-T cell activation remains a significant challenge for the effective clinical application of CAR-T therapeutics. In fact, CAR-T cells directed against MUC1 and PSCA are not able to completely eliminate solid tumors, and tumor cells expressing low densities of the target antigen are the main reason for tumor escape (43). Additionally, solid tumors display substantial heterogeneity of phenotypes and target antigens. In an EGFRvIII-targeted CAR-T first-in-human study, post-therapeutic biopsies of glioblastoma revealed that the levels of EGFRvIII antigen expression reduced after treatment compared with pre-treatment tumors in 71% of the patients (31, 32). The inability of the CAR-T cells to detect and eliminate antigen-low tumor cells may be related to the failure of treatment in this study.

The two additional obstacles in achieving a beneficial therapeutic effect using CAR-T therapy on solid tumors are the poor trafficking to both tumor sites and to the tumor microenvironment (TME) (44, 45). Unlike the situation in blood cancer, CAR-T cells cannot effectively traffic from the blood into the solid tumor mass. The chemokine receptor can be co-expressed in CAR-T cells to increase the trafficking of CAR-T cells to solid tumor sites. Several groups have reported that CAR-T cells, which express the chemokine receptor CCR2b, increase the tumor infiltration and antitumor activity of CAR-T cells (46, 47). Another chemokine receptor combined with CAR-T cells is CXCR2 (48, 49), which has been shown to improve the homing of CAR-T cells to the tumor. CARs expressing IL12β p40 subunit to produce IL-23 upon activation, which activates STAT3 signaling to promote proliferation, showed improved activities in mice (50, 51). The chemokine receptors co-expressed in CAR-T cells need to be selected according to the specific tumor types because different tumor types have different expression patterns of chemokines (52–55). Thus, more chemokine receptors must be found and validated for solid tumors. Alternatively, locoregional delivery of CAR-T cells and repeat treatment may help improve the therapeutic effect of CAR-T therapy. Donovan et al. identified EPHA2 as a tumor antigen for medulloblastoma and designed trivalent CAR-T cells (EPHA2, HER2, IL13Rα2) to perform preclinical studies and validated that intrathecal delivery of the trivalent CAR-T could be an effective treatment for metastatic medulloblastoma (56). Therefore, cancer-type-specific CAR-T delivery and transplantation may also help overcome the obstacle of poor trafficking.

The TME has numerous suppressive immune cells and molecular factors, which impair the cytotoxic function of CAR-T cells (57, 58). Overcoming the immunosuppressive effects of the TME is the main focus of CAR-T therapy research in solid tumors (59). Several strategies have been proposed for CAR-T cells to resist immunosuppression of the TME. Suryadevara et al. reported that the prevention of lymphocyte-specific tyrosine kinase (Lck) binding in the CD28 domain eliminated the secretion of IL-2 from CAR-T cells, and hence, CAR-T cells achieved resistance to regulatory T cells (Tregs) (60). Mohammed et al. showed that CAR-T cells expressing 4/7 inverted cytokine receptors could reverse the immunosuppressive signal from IL-4 to the signal of proliferation (61). Yamamoto et al. found that the apoptosis-inducing ligand FasL (CD95) is overexpressed within the TME of many human cancer types and designed T cells to prevent Fas-induced apoptosis (44). Rafiq and colleagues reported that CAR-T cells expressing PD-1-blocking single-chain variable fragment (scFv) achieved resistance to PD-L1 inhibition (62). However, many inhibitory factors exist in the TME: a strategy to resolve one inhibitory factor may succeed in mouse models, while more efforts may be required to achieve expected therapeutic effects in clinical trials.

CAR products cannot be easily expanded under good manufacturing practice (GMP) conditions which limits their application as “off-the-shelf” products for patients. On the other hand, CAR-modified NK cells may help solve this problem, owing to their tolerable safety profile and low possibility of triggering graft-versus-host disease upon the allogeneic infusion of CAR-modified NK cells without the constraint of autologous cells. An *ex-vivo* expansion method of NK cells from CD34^+^ umbilical cord blood is now an option for a clinical-grade protocol for adoptive immunotherapy. Induced pluripotent stem cells (iPSCs) are also sources of functional NK cells. The iPSC-derived NK cells can be expanded 10^2^- to 10^3^-fold with a panel of cytokines, including IL-3, IL-7, IL-15, SCF, and Flt3L, without exogenous stromal cells (63), which is promising for future development. A study comparing the cytotoxicity of NK cells isolated from peripheral blood mononuclear cells and iPSCs for killing ovarian cancer cells (64) indicated that both NK cell populations had a significant effect on the killing of cancer cells. Gene editing of iPSCs with CRISPR/Cas9 could also improve the therapeutic potential of iPSC-derived NK cells (iPSC-NKs). A study by Zhu et al. revealed that knocked-out cytokine-inducible SH2-containing protein (encoded by the gene CISH) developed CISH^−/−^ iPSC-NKs, showing increased expansion and cytotoxic effects on tumor cell lines including ovarian tumor cells (65). The expression of CARs can further boost the antitumor activity of effector NK cells. Another study showed that CAR-iPSC-NK cells inhibited ovarian tumor growth more effectively compared with iPSC-NK cells (66).

### Improvements in CAR Design

Currently, CAR-T cells used in preclinical or clinical trials are mostly based on 4-1BB or CD28, but these CAR designs have some downsides, such as cytokine release syndrome and macrophage activation syndrome (67, 68). Guedan et al. reported that CAR-T cells with ICOS and 4-1BB co-stimulation had an optimal antitumor activity and persistence *in vivo* (67). In contrast to CAR-T, TILs or TCR-engineered T cells that rely on TCR signaling have reported low rates of adverse events (68). Helsen et al. developed a new chimeric receptor termed TAC (T-cell antigen coupler) which transduces signals through the native TCR, and TAC-engineered T cells display both enhanced *in-vivo* antitumor efficacy and decreased off-tumor toxicity compared to the first- and second-generation CARs (68). Another chimeric receptor utilizing native TCR for transducing signal is TRuC (T-cell receptor fusion construct). TRuC-T cells have shown the capacity for trafficking to tumors and long-term functional persistence in a model (69).

Whether using a typical CAR that is based on co-stimulator 4-1BB/CD28 or the TAC/TRuC that hijacks the endogenous TCR subunits to form a CAR complex, CD3ζ is required for the activation of these artificial receptors. However, in the TME, CD3ζ expression is always downregulated by arginine exhaustion with arginase (70, 71); hence, CARs based on CD3ζ may have a disadvantage in treating solid tumors. Recently, a natural multichain immunoreceptor design showed potential antitumor activity for solid tumors. Wang and colleagues (72) developed a multichain CAR design that is not based on CD3ζ but rather on KIR/DAP12. KIR-CAR/DAP12 expression in T cells is not affected in solid tumors, while CARs based on CD3ζ are not fully expressed in solid tumors. Moreover, Dap12-based CAR-T cells showed resistance to tumor-induced hypofunction and enhanced antitumor activity compared to CD3ζ-based CAR-T cells. Currently, Dap12-based CAR-T immunotherapy is being tested in clinical trials by Wang and colleagues, and the preliminary results show that one ovarian cancer patient achieved SD over 16 months and the ascites of two patients significantly decreased along with effective CAR-T cell expansion both in blood and ascites (data not published). In 2019, another Dap12-based CAR design was reported (73), which was TREM1/Dap12-based and showed faster tumor eradication than BBζ CAR-T cells in mice.

In conclusion, because of the suppression of CD3ζ within TME, Dap12-based multichain CAR showed advantages when applied to solid tumors.

### Future Directions of CAR-T Therapy

Current clinical trials show that TAAs for CAR-T therapy in solid tumors are not specific to tumor cells. In fact, it is difficult to find targets highly and uniquely expressed in solid tumors. Thus far, B7-H3 and DR4/DR5 have been established as potential targets for treating solid tumors. In addition to antigen identification, overcoming the dysfunction of CAR-T cells in the TME is the most important challenge for improving therapeutic effects in solid tumors. Some strategies have been proposed to enhance the function of CAR-T in TME, but these strategies need to be clinically validated. As mentioned earlier, mutations in the CD28 domain can improve CAR-T resistance to Treg cells, implying that CAR designs currently in use are not perfect. The bottleneck of CAR-T therapy for solid tumors may be addressed by changing the design of CARs to optimize the T-cell signal transduction pathway (74), especially the endoplasmic domain of CAR.

Most research groups focus on target switching or new antibody generation. However, barriers to CAR-T therapy against solid tumors cannot easily be overcome using this strategy; hence, artificial receptors approaching two destinations, tumor recognition and immune cell activation, need to be designed. Just mimicking TCR may not serve the purpose. Other immune cell-activating receptors may also be mimicked. Single-chain artificial receptors can be substituted by multiple-chain receptors with an architecture closer to that of natural activating receptors. Also, antigen-dependent control of CAR may enhance its effectiveness. For instance, Hernandez-Lopez et al. engineered a two-step positive-feedback circuit attempting to allow cytotoxic T cells to discriminate targets upon the density of TAAs (75). Jan et al. put efforts into generating lenalidomide-controlled reversible ON- and OFF-switch CARs, under which the system CARs are designed to split under the ON-mode and be degraded under the OFF-mode (76). In addition, advanced nanomaterial-based CAR deliveries are also in development (77). With these new designs, the therapeutic effects of CARs against solid tumors may be improved, and TME suppressive effects may also be avoided during CAR-T cell expansion and functionality realization.

CAR-T/immune cell survival and tumor homing have gained increasing attention. Antiregulatory signal antibodies and homing signals have been tested with the so-called “travel trailer style” design to provide more T/immune cell growth factors, which will be validated soon in clinical trials. In addition to the molecular level design, combining CAR-modified immune cell therapies themselves or other treatments, such as immune checkpoint blockade or traditional cancer treatment, are worthy of being tested in the near future (78).

## Concluding Remarks

After comparing the regulations from different countries on cell therapy from several sources including engineered cells and products that recruit, mobilize, and activate human cells in treating human malignant tumors, ideally, CAR-T should select the tumor-specific antigens that have no on-target/off-tumor toxicities upon more advanced research. An “off-the-shelf” product for cancer treatment should be well considered for wide application in clinical practice for its ease of availability and large-scale expansion. Most importantly, cell therapy is expected to have a positive impact on the treatment of solid tumors (**Figure 1**). Thus, supportive and reasonable guidelines from related authorities worldwide may ensure promising, beneficial, and favorable results.

## Author Contributions

All authors listed have made a substantial, direct, and intellectual contribution to the work and approved it for publication.

## Funding

This work was supported by the Chinese Academy of Medical Sciences Innovation Fund for Medical Sciences (Platform Improvement of Clinical Trial Capability 2020-I2M-2-007) (recipient: NL), Chinese Academy of Medical Sciences (grants 2019XK320068) (recipient: NL), Beijing Municipal Science and Technology Commission (International Pharmaceutical Clinical Research and Development Platform 2015) (recipient: NL), and Corbett Estate Fund for Cancer Research (62285-531021-41800; 62285-531021-51800; 62285-531021-61800; 62285-531021-71800) (recipient: EW).

## Conflict of Interest

The authors declare that the research was conducted in the absence of any commercial or financial relationships that could be construed as a potential conflict of interest.

## Publisher’s Note

All claims expressed in this article are solely those of the authors and do not necessarily represent those of their affiliated organizations, or those of the publisher, the editors and the reviewers. Any product that may be evaluated in this article, or claim that may be made by its manufacturer, is not guaranteed or endorsed by the publisher.

## Glossary

**Table:**

CARs	chimeric antigen receptors, are engineered receptors, which graft an arbitrary specificity onto an immune effector cell (T cell). Typically, these receptors are used to graft the specificity of a monoclonal antibody onto a T cell, with the transfer of their coding sequence facilitated by retroviral vectors. The receptors are called chimeric because they are composed of parts from different sources. CARs are under investigation as a therapy for cancer, using a technique called adoptive cell transfer. T cells are extracted from a patient and modified so that they express receptors specific to the patient&rsquo;s particular cancer. CARs are synthetic molecules composed of an extracellular domain, a transmembrane domain, and an intracellular signaling domain. The extracellular component is an scFv, which recognizes and binds specific TAAs. The transmembrane domain is typically derived from CD8 molecules, and the intracellular signaling domain consists of CD3&zeta; and one or two co-stimulatory domains (CD28 and/or 4-1BB).
TCR	T-cell receptor, is a protein complex found on the surface of T cells or T lymphocytes that is responsible for recognizing fragments of antigen as peptides bound to major histocompatibility complex (MHC) molecules. The binding between TCR and antigen peptides is of relatively low affinity and is degenerate: that is, many TCRs recognize the same antigen peptide and many antigen peptides are recognized by the same TCR.
TILs	tumor-infiltrating lymphocytes, which include B cells, T cells, and natural killer (NK) cells, form an important component in antitumor immune responses.
DCs	dendritic cells, are any of the various white blood cells that have long projections from the cell body and function in the immune response by taking in and processing antigens and presenting them to T cells in lymph nodes, thus activating the T cells. Immature dendritic cells are found chiefly in the skin and mucosal surfaces.
NK	natural killer cells, are large granular lymphocytes that do not express markers of either T- or B-cell lineage. These cells kill target cells through antibody-dependent cell-mediated cytotoxicity. NK cells can also use perforin to kill cells in the absence of antibody
scFv	single-chain variable fragment, consists of a variable heavy (VH) and a variable light (VL) antibody chains linked with a peptide linker.
GPCRs	Chemokine receptors are a superfamily of G-protein coupled receptors that control immune cell behavior *via* the promotion of chemotaxis, cell adhesion, and mediator release.
TME	tumor microenvironment, is the environment surrounding a tumor and consists of protean components, such as immune cells, blood or lymphatic vessels, fibroblasts, endothelial cells, pericytes, and extracellular matrix (ECM); the tumor closely interacts with its TME, which contributes to the generation of therapy resistance, metastasis, and immune escape.
