# Supplementary material for: Clinical Investigations of CAR-T Cell Therapy for Solid Tumors
Source: Front Immunol. 2022 Jul 18;13:896685. doi: 10.3389/fimmu.2022.896685 (PMC9339623; doi:10.3389/fimmu.2022.896685)
Supplement: Supplementary file 1 [file DataSheet_1.doc]

INFORMA ([https://pharma.id.informa.com](https://pharma.id.informa.com/)). Pharmaprojects and Trialtrove are a part of the Citeline suite of products maintained by Informa Pharma Intelligence [1]. Citeline organizes vast quantities of data with human intelligence to collate and index information available in the public domain regarding drugs in development and clinical trials. Pharmaprojects and Trialtrove index information from over 40,000 public sources, including Clinicaltrials.gov and Gene Therapy Clinical Trials Worldwide, among many other sources [2]. Pharmaprojects has been tracking drug development for over 40 years and Trialtrove has been tracking clinical trial information for over 16 years. Both products have been cited in numerous peer-reviewed publications [3].

Using the following key words for the inquiry: [(Therapeutic Class is Cellular therapy, chimaeric antigen receptor) OR (Therapeutic Class is Cellular therapy, other) OR (Therapeutic Class is Cellular therapy, stem cell) OR (Therapeutic Class is Cellular therapy, T cell receptor) OR (Therapeutic Class is Cellular therapy, tumour-infiltrating lymphocyte)] AND [(Disease is not Oncology: Unspecified Hematological Cancer) AND (Disease is not Oncology: Supportive Care) AND (Disease is not Oncology: Leukemia, Acute Lymphocytic) AND (Disease is not Oncology: Leukemia, Acute Myelogenous) AND (Disease is not Oncology: Leukemia, Chronic Lymphocytic) AND (Disease is not Oncology: Leukemia, Chronic Myelogenous) AND (Disease is not Oncology: Myelodysplastic Syndrome) AND (Disease is not Oncology: Myeloproliferative Neoplasms) AND (Disease is not Oncology: Multiple Myeloma) AND (Disease is not Oncology: Lymphoma, Non-Hodgkin's) AND (Disease is not Oncology: Lymphoma, Hodgkin's) AND (Disease contains oncology) AND (Drug Disease contains oncology) OR (Drug Disease contains cancer) OR (Drug Disease contains solid cancer)] AND (Actual Start Date is from 2011/01/01 to 2021/01/01).

Reference:

1. <https://pharmaintelligence.informa.com/products-and-services/data-and-analysis/citeline>
2. https://pharmaintelligence.informa.com/products-and-services/data-and-analysis/pharmaprojects
3. https://pharmaintelligence.informa.com/products-and-services/data-and-analysis/trialtrove
